# Immunomodulation of Host Chitinase 3-Like 1 During a Mammary Pathogenic *Escherichia coli* Infection

**DOI:** 10.3389/fimmu.2018.01143

**Published:** 2018-05-28

**Authors:** Koen Breyne, Jonas Steenbrugge, Kristel Demeyere, Chun Geun Lee, Jack A. Elias, Wolfram Petzl, David G. E. Smith, Pierre Germon, Evelyne Meyer

**Affiliations:** ^1^Laboratory of Biochemistry, Department of Pharmacology, Toxicology and Biochemistry, Faculty of Veterinary Medicine, Ghent University, Merelbeke, Belgium; ^2^Division of Biology and Medicine, Warren Alpert School of Medicine at Brown University, Providence, RI, United States; ^3^Clinic for Ruminants with Ambulance and Herd Health Services, Centre for Clinical Veterinary Medicine, Ludwig-Maximilians-University of Munich, Munich, Germany; ^4^Institute of Biological Chemistry, Biophysics and Bioengineering, Heriot-Watt University, Edinburgh, United Kingdom; ^5^INRA UMR 1282 Infectiologie et Santé Publique (ISP), Université François Rabelais de Tours, Nouzilly, France

**Keywords:** chitinase 3-like 1, *Escherichia coli*, bovine mastitis, immunomodulation, mouse mammary gland infection model

## Abstract

Chitin is a *N*-acetyl-d-glucosamine biopolymer that can be recognized by chitin-binding proteins. Although mammals lack chitin synthase, they induce proteins responsible for detecting chitin in response to bacterial infections. Our aim was to investigate whether chitinase 3-like 1 (CHI3L1) has a potential role in the innate immunity of the *Escherichia coli* (*E. coli*) infected mammary gland. CHI3L1 protein was found to be secreted in whey of naturally coliform-affected quarters compared to whey samples isolated from healthy udders. In addition, gene expression of CHI3L1 was confirmed in udder tissue of cows experimentally infected with a mammary pathogenic *E. coli* (MPEC) strain. Despite the known anatomical differences, the bovine udders’ innate immune response was mimicked by applying an experimental mouse model using MPEC or non-MPEC isolates. The effect of CHI3L1 expression in the murine mammary gland in response to coliform bacteria was investigated through the use of CHI3L1^−/−^ mice as well as through treatment with either a pan-caspase inhibitor or chitin particles in wild-type mice. The local induction of CHI3L1 postinfection with different *E. coli* strains was demonstrated to be independent of both bacterial growth and mammary interleukin (IL)-8 levels. Indeed, CHI3L1 emerged as a regulator impacting on the transcytosis of Ly6G-positive cells from the interstitial space into the alveolar lumen of the mammary tissue. Furthermore, CHI3L1 was found to be upstream regulated by caspase activity and had a major downstream effect on the local pro-inflammatory cytokine profile, including IL-1beta, IL-6, and RANTES/CCL5. In conclusion, CHI3L1 was demonstrated to play a key role in the cytokine and caspase signaling during *E. coli* triggered inflammation of the mammary gland.

## Introduction

Chitin is a prominent polymer present within the cuticle of arthropods, plays a crucial part of cephalopods and the cell wall of fungi, but is absent in mammals (1–3). Chitin consists of a repetitive linear chain of β-1,4 linked *N*-acetyl-d-glucosamine units. As potential microbial-associated molecular pattern, mammals have the ability to either bind or process chitin by using the number-18 glycoside hydrolase (GH18) family, which can be divided into two subfamilies: chitinase-like proteins (CLPs), which bind chitin but do not possess chitinolytic activity and chitinases, which bind chitin and subsequently hydrolyze their substrate (4).

Mammals use the chitin recognition machinery to protect themselves against pathogenic chitinous species (5, 6). Members of the GH18 family detect chitin in pathogenic organisms and trigger expression and activation of mammalian chitinase(-like) proteins, which have been implicated in the inflammatory response against these organisms (7). Interestingly, mammalian CLPs are also induced in response to chitin-lacking bacterial pathogens (8) and even to non-infectious diseases like cancer (9–14). In response to lung infections or bone marrow-derived macrophages, chitinase 3-like 1 (CHI3L1) participates in the immune defense and bacterial clearance through inhibition of caspase-1-dependent macrophage pyroptosis (15, 16). These observations imply that mammalian chitinase(-like) proteins are likely to play a key role of the hosts’ (innate) immune response (17, 18). However, the underlying molecular mechanism in which these proteins are involved remains poorly understood.

Chitinase(-like) proteins have been analyzed in extracellular fluids, such as milk (19, 20). In the mammary tissue, they have been implicated in different processes such as mammary involution, tumor development, epithelial apoptosis, and inflammation (21–23). The mammary inflammatory response is of crucial importance for dairy cattle to cope with udder infections (24) and is one of the three most important factors affecting cow replacement in dairy herds. CLPs have been found in the udder secretions collected during the non-lactating period, in colostrum but not in normal milk (25, 26). Interestingly, however, a few proteome studies reported elevated levels of CLPs in normal milk upon mastitis (27–31). Yet, the biological role of these proteins during bovine mastitis remains unknown so far.

Our main aim was to investigate the potential role of CHI3L1 during coliform mastitis and assess its contribution to the innate immune response in the infected mammary gland. For this purpose, the levels of the CHI3L1 protein were assessed in whey from healthy and naturally mastitis-affected cows by Western blot as well as mRNA expression in udder tissue of cows experimentally infected with *Escherichia coli*. Subsequently, a bovine coliform mastitis was simulated through an established murine mastitis model using *E. coli* isolates from clinical cases of bovine mastitis. The concentration of CHI3L1 was determined together with key infection or inflammation parameters at the local level (bacterial load, neutrophil influx, interleukin (IL)-8 and IL1beta, IL-6, RANTES/CCL5 levels). Third, in this validated murine mastitis model, the effects of CHI3L1 induction on up-/or downstream molecules were evaluated using either CHI3L1-deficient mice or different treatments that have been shown to influence CHI3L1 in other mouse models, such as pan-caspase inhibition (15) and chitin treatment (10).

## Materials and Methods

### Preparation of Whey Protein Fraction From Milk Samples

Bovine milk samples were obtained from naturally coliform-affected quarters (*n* = 6) and from healthy quarters (*n* = 3). Milk was centrifuged at 1,500 *g* for 20 min at 4°C to discard the fatty layer. The skimmed milk was then ultra-centrifuged for 1 h at 53,300 *g* at 4°C to pellet casein micelles. The protein concentration in the samples was determined spectrophotometrically (Genesys 10UV, Analis) using Bradford Protein Assay.

### Western Blotting Analysis

Bovine whey proteins were loaded in equal amounts (72 µg protein per lane) on a 8–16% gradient polyacrylamide Amersham ECL Gel (GE Healthcare). After separation, proteins were transferred to a 0.45-µm nitrocellulose transfer membrane (Biorad). To detect bovine CHI3L1, the membranes were blocked in Tris-Buffered Saline (TBS) supplemented with 0.1% Tween-20 (v/v), 0.1% bovine serum albumin, and 4% (w/v) non-fat dry milk and incubated overnight at 2–8°C on a platform shaker with a rabbit anti-bovine CHI3L1 polyclonal/monoclonal antibody (1.5 mg ml^−1^), kindly provided by Tom Wheeler, AgResearch, diluted 1:1,000 in TBS supplemented as described (31). Next, the membranes were washed in TBS (3 times for 5 min), and membranes were probed using a donkey anti-rabbit IgG Horseradish Peroxidase-conjugated polyclonal antibody (1 mg ml^−1^, Thermo Fisher Scientific, diluted 1:20,000) for 60 min at room temperature (RT). The resulting CHI3L1 bands were visualized with Western Lightning™ Chemiluminescence Reagent Plus (PerkinElmer) on autoradiographic films (Amersham).

### RT-qPCR Analysis of CHI3L1 Transcripts in Mammary Tissue From Infected Cows

RNA was extracted from mammary gland tissue of German Holstein Friesian cows (*n* = 5) in mid-lactation, experimentally infected with *E. coli* strain 1303 as described previously (32) using Trizol (Invitrogen). Experimental infection of animals was conducted at the Clinic for Ruminants (Munich, Germany) with the approval of the ethics committee of the regional government of upper Bavaria, Germany (No. 55.2-1-54-2531-108-05). Total RNA (1 µg) was reverse transcribed to cDNA using the iScript™ Reverse Transcription Supermix (Biorad). Quantitative PCR was then performed using the iQ SYBR Green Supermix (Biorad), using the primers listed in Table 1. Briefly, after normalization using the expression of three reference genes (ActB, PPIA, 18S RNA), expression of each gene was calculated using the Genex macro (Biorad) relative to the values obtained from uninfected cows arbitrarily set to 1.

**Table 1:** Primers used for RT-qPCR.

Gene	Forward primer sequence 5′–3′	Reverse primer sequence 5′–3′
IL-1beta	CTCTCACAGGA AATGAACCGAG	GCTGCAGGGTG GGCGTATCACC
IL-6	TGCTGGTCTTCT GGAGTATC	GTGGCTGGAGT GGTTATTAG
IL-8	TGAAGCTGCAG TTCTGTCAAG	TTCTGCACCCAC TTTTCCTTGG
Chitinase 3-like 1	TCTGTTGGAGGA TGGAACTTCG	TGGCACCGACTT GATGAAAG
18S	CGGGGAGGTAGT GACGAAA	CCGCTCCCAAGA TCCAACTA
ACTB	ACGGGCAGGTCA TCACCATC	AGCACCGTGTTG GCGTAGAG
PPIA	TCCGGGATTTATGT GCCAGGG	GCTTGCCATCCAA CCACTCAG

### *E. coli* Murine Intramammary Infection Model

The *in vivo* effect of the clinical bovine mastitis isolates *E. coli* O32:H37 strain P4 and *E. coli* O70:H32 strain 1303, alone or in combination with a treatment was evaluated using a murine intramammary infection model. Approval number EC2016/56 was obtained from the Animal Ethics committee (Faculty of Veterinary Medicine, Merelbeke, Belgium). In brief, C57BL/6 (*n* = 28), CHI3L1^−/−^ (also C57BL/6 background, *n* = 6), and CD-1 (*n* = 33) dams were utilized 12–14 days (d) after birth of the offspring. Wild-type mice were purchased from Envigo and CHI3L1^−/−^ mice were obtained from Prof. Jack A. Elias (Brown University). The CHI3L1^−/−^ mice were originally generated in C57BL6/J cells and have been maintained on a C57BL6/J strain of mice (33). All inoculations were performed 2 h post-weaning under anesthesia. A mixture of oxygen and isoflurane (2–3%) was used for inhalational anesthesia of the lactating mice combined with a long-acting analgesic buprenorphine, intraperitoneally injected (at 10 µg kg^−1^ Vetergesic^®^, Patheon UK Ltd.). A syringe with a 32-gauge blunt needle (Thiebaud Biomedical Devices) was used to inoculate both L4 (on the left) and R4 (on the right) glands of the fourth abdominal mammary gland pair with approximately 1,000 colony forming units (CFU) of *E. coli* resuspended in phosphate buffered saline (PBS). Each orifice was exposed by a small cut at the near end of the teat and 100 µl of the inoculum was injected slowly through the teat canal. When necessary, chitin particles were instilled into the mammary gland of anesthetized mice using the desired dose (approximately 10^6^ particles in 200 µl of PBS per gland) at 4 h after bacterial inoculation. To test the effect of local caspase inhibiton on CHI3L1 induction, Z-VAD-fmk (Abcam) was dissolved into DMSO and diluted in PBS to a concentration of 2.5% DMSO. When required, the R4 mammary gland was used for an intramammary injection (250 µl) with Z-VAD-fmk (corresponding to 350 mg kg^−1^) while L4 was used for a sham (2.5% DMSO) treatment of *E. coli* P4 infected mammary glands at 4 h p.i. (34).

24 h p.i., all mice were sedated by administering a mixture of ketamine (at 100 mg kg^−1^ Anesketin, Eurovet Animal Health BV) with xylazine (at 10 mg kg^−1^; Xylazini Hydrochloridum, Val d’Hony-Verdifarm) intraperitoneally and subsequently euthanized by cervical dislocation. The mammary glands (two per mouse), which are physiologically separated and thus were considered as individual samples, were harvested and divided for the different analyses. For CFU and protein assays, mammary gland tissue was weighed and homogenized using a tissueruptor (QIAGEN Benelux BV). Bacterial CFU counts were obtained by standard plating on tryptic soy agar (Oxoid).

### H&E Histology, TUNEL Assay, and Immunohistochemistry

Two mammary glands per condition were fixed in buffered 3.5% formaldehyde for 24 h at RT and embedded in paraffin wax. Sections were deparaffinized, hydrated, and stained with hematoxylin and eosin. Subsequently, sections were rehydrated and mounted on a glass-slide and covered with a coverglass.

Apoptosis was quantitated in the mammary glands treated with Z-VAD-fmk and PBS-diluted DMSO (sham) by using the terminal deoxynucleotidyl transferase-mediated deoxyuridine triphosphate nick-end labeling (TUNEL) assay. This specific assay uses terminal deoxynucleotidyl transferase to attach fluorescein labeled deoxyuridine triphosphate to free 3′-OH DNA ends. Sections of 5 µm were prepared from paraffin-embedded tissues using a microtome and placed on APES (3-Aminopropyltriethoxysilane)-coated glass slides. The sections were deparaffinized in xylene and dehydrated in ethanol. The sections were incubated with a commercial proteinase K kit (DAKO, Danmark) diluted in TE-buffer (Tris-EDTA; pH 8) for 15 min at RT. After rinsing the specimen twice with PBS, the sections were processed following the instruction of a commercial kit (*In Situ* Cell Death Fluorescein Detection Kit; Roche Applied Science, Shanghai, China). Sections were counterstained with Hoechst to visualize the nuclei. The TUNEL-positive cells were identified as green fluorescein staining nuclei. The number of TUNEL-positive cells was counted in 20 random microscopic fields (40×).

Immunohistochemical stainings for Ly6G were performed on 2- to 3-µm paraffin sections. The tissue slides were deparaffinized and incubated with citrate buffer (pH 6, containing 10 mM tri-sodium citrate and 0.05% Tween-20) in a pressurized decloacking chamber at 95°C for 30 min. Blocking of endogenous peroxidase activity was performed by incubating the slides in a mixture of 3% H_2_O_2_ in methanol for 10 min. Primary rat anti-mouse Ly6G antibody (clone 1A8, BioLegend, diluted 1:1,000 in antibody diluent from Dako) was applied on the tissue slides for 1 h at RT. Secondary rat-on mouse HRP-Polymer (Biocare Medical) was then applied for 30 min at RT. Final detection of antibody staining was established by incubation of the tissue slides in a buffer containing 3,3′-diaminobenzidine for 10 min at RT. All incubation steps were performed in a humidified chamber on an orbital shaker. Rinsing of the slides in between incubation steps was performed with TBS. Quantification of immunohistochemical stainings was performed using Image J (3 color split and automatic cell counting).

### Preparation of Chitin Particles

A chitin particle emulsion was prepared as previously described with minor alterations (35). Briefly, chitin powder (Sigma-Aldrich) was suspended at 10 mg ml^−1^ in PBS (Thermo Fisher scientific) and sonicated with an ultrasonic probe sonifier 450 (VWR, Branson Ultrasonics). The solution was filtered through a 10-µm strainer (pluriSelect), pelleted after centrifugation (250 *g*, 10 min) and finally suspended in PBS. The size and amount of the chitin particles in the obtained pellet was verified by flow cytometry (FACSCanto, BD Biosciences) using BD Trucount beads (8 µm) and Cytometric Bead Array beads (3 µm). The size of the chitin particles in the gained suspension ranged between 3 and 8 µm.

### Cytokine Multiplex Assay, Chitinases Activity Assay, and CHI3L1 ELISA

One hundred microliters of mouse mammary gland homogenate were mixed with 300 µl lysis buffer supplemented with protease inhibitors (200 mM NaCl, 10 mM Tris–HCl pH 7.4, 5 mM EDTA, 1% Nonidet P-40, 10% glycerol, 1 mM oxidized l-glutathion, 100 µM PMSF, 2.1 µM leupeptin, and 0.15 µM aprotinin) to extract cellular proteins. The suspensions rested overnight at −20°C, were centrifuged at 12,250 *g* for 1 h and finally the supernatant was centrifuged for another 30 min to precipitate the pellet. Protein concentration of the lysate samples was determined as previously described (36). Cytokine quantification (ProcartaPlex from eBioscience), chitinase assays (Sigma), and CHI3L1 ELISA (Biotechne) on the mammary gland lysates were all performed according to the manufacturer’s instructions. Briefly, chitinase activity assay determines exo- and endochitinase activity through the production of fluorogenic products. The exochitinase activity releases one *N-*acetyl-d-glucosamine residue from the (non)reducing end, and can be measured with an artificial substrate such as 4-methylumbelliferyl (MU) *N,N*′-diacetyl-β-d-chitobiose, while the endochitinase activity prefers longer *N-*acetyl-d-glucosamine chains and can, therefore, be measured with substrates such as 4-MU *N,N′,N′′-*triacetyl-β-d-chitotriose (11).

### Statistical Analysis

Statistical analysis was performed using Prism (GraphPad) and SPSS (IBM Analytics). Data were checked for normality and an unpaired Student’s *t*-test was used to determine whether differences between groups were statistically significant (*P* < 0.05).

## Results

### CHI3L1 Is Present in Whey From Naturally Coliform-Infected Udder Quarters and Expressed in Experimentally *E. coli*-Infected Udder Quarters

**Figure 1:**
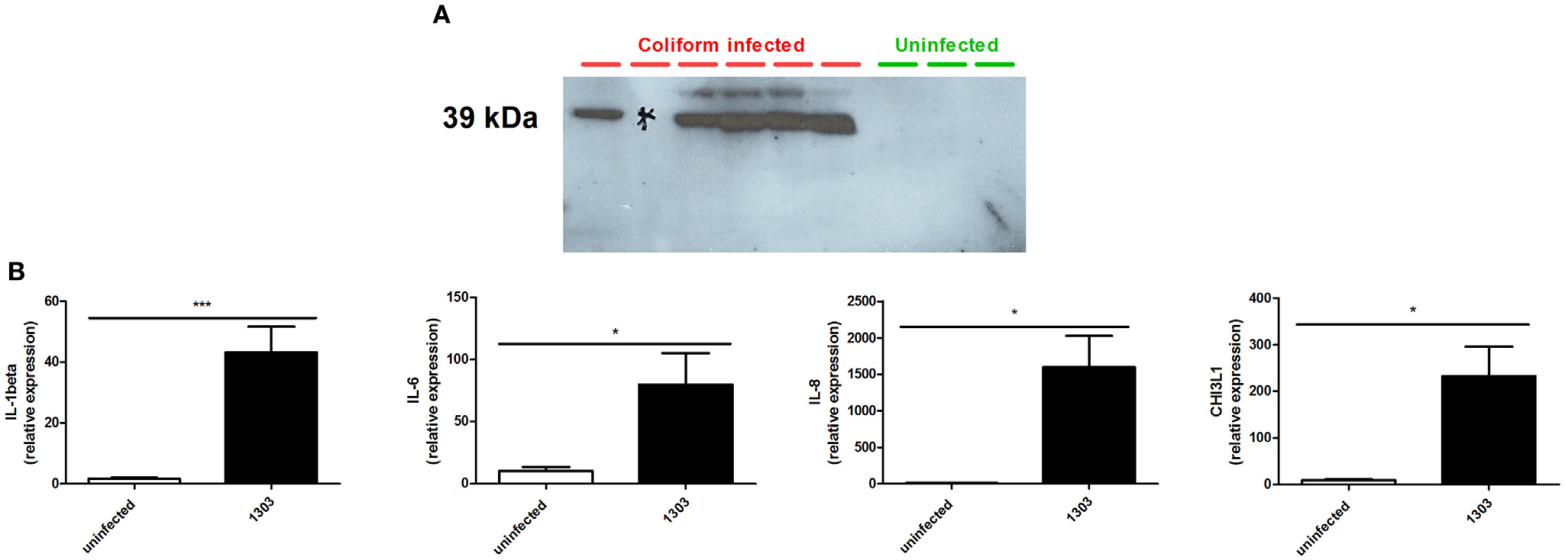
Chitinase 3-like 1 (CHI3L1) in coliform-infected bovine samples. **(A)** Bovine CHI3L1 protein detection in whey isolated from naturally coliform-infected cows (*n* = 6) compared to whey isolated from healthy quarters (*n* = 3). **(B)** interleukin (IL)-1beta, IL-6, IL-8, and CHI3L1 expression in *Escherichia coli* 1303 experimentally infected cows (*n* ≥ 4). Data are presented as means ± SEM, indicating significant differences with *(*P* < 0.05).

Whey obtained from milk samples of dairy cows diagnosed with coliform mastitis (*n* = 7) were analyzed for the presence of CHI3L1. Rather than using an uncharacterized bovine ELISA, a previously characterized primary antibody against bovine CHI3L1 (31) was used for CHI3L1 detection in coliform-infected samples (*n* = 6). In five out of six coliform whey samples, it showed a strong signal by Western blotting while this 39-kDa protein band was absent in all whey samples obtained from bacteriologically negative quarter milk (*n* = 3) (Figure 1A; Figure S1 in Supplementary Material).

Following experimental infection of cows (different animals than those used for Western blotting analysis) with the bovine *E. coli* 1303 mammary pathogenic *E. coli* (MPEC) strain, IL-1beta, IL-6, IL-8, and CHI3L1 mRNA expression levels in these udder quarters were compared with those from uninfected quarters from the same cows (internal controls) and quarters of uninfected cows (external controls, data not shown). Compared to expression levels in uninfected quarters, *E. coli* 1303 infection significantly induced IL-1beta, IL-6, and IL-8 concomitantly with CHI3L1 in these infected quarters (Figure 1B).

In addition to CHI3L1 expression, chitinolytic activity is also of interest during coliform mastitis. Whey samples from coliform-affected quarters showed significantly higher exochitinase and endochitinase activities compared to those from healthy quarters (Figure S2A in Supplementary Material).

### CHI3L1 Is Induced Independently of the Mammary Bacterial Loads and IL-8 Levels in a Murine Mastitis Model

**Figure 2:**
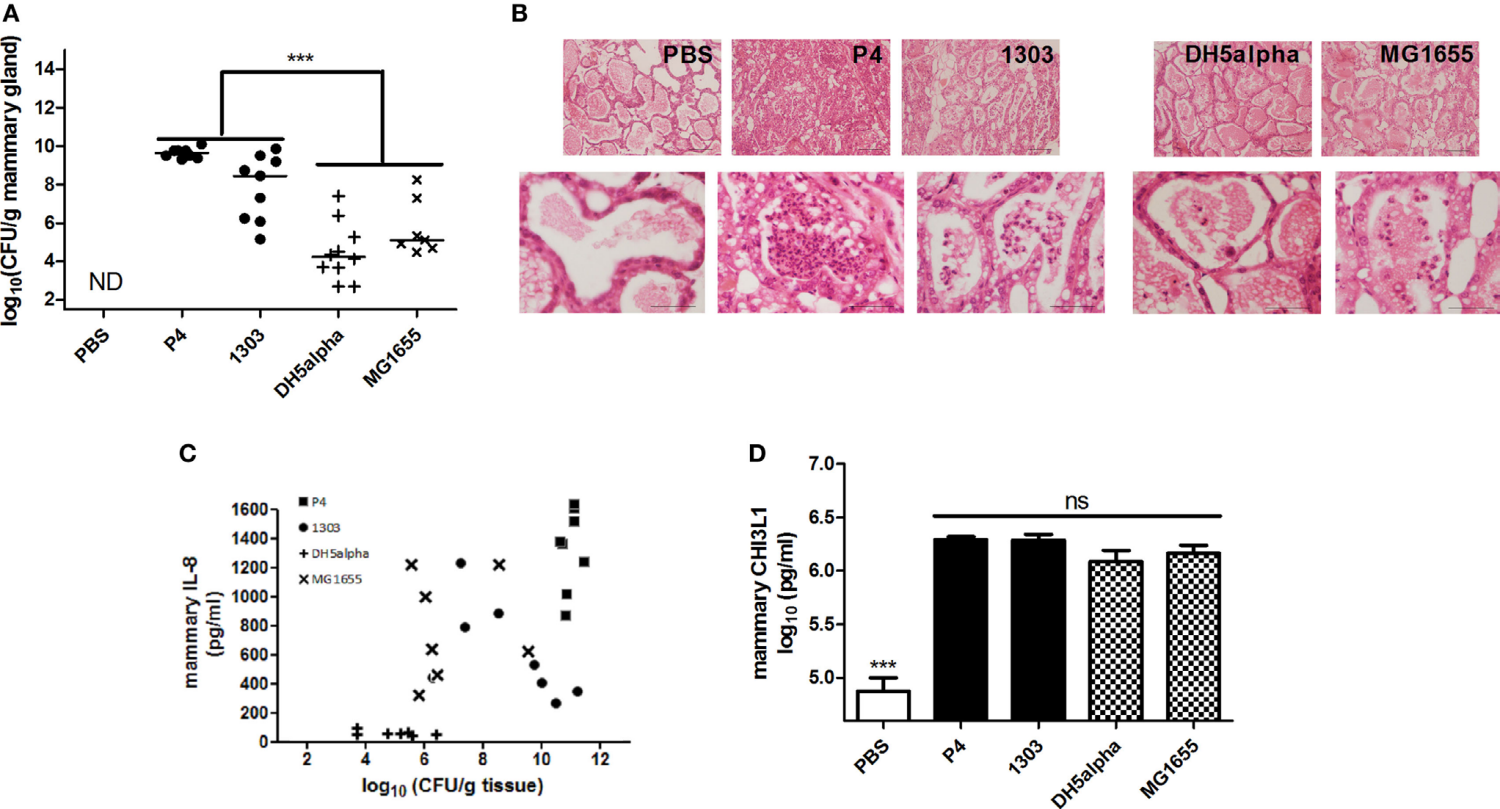
Mammary chitinase 3-like 1 (CHI3L1) during mammary pathogenic *E. coli* (MPEC) and non-MPEC infections in the mouse mastitis model. **(A)** Bacterial loads in the mammary gland at 24 h p.i. with MPEC strains *Escherichia coli* P4 and 1303 compared to non-MPEC strains *E. coli* K12 strains DH5alpha and MG1655, and phosphate buffered saline (PBS) controls (*n* ≥ 8). **(B)** Histology of mammary tissue at 24 h p.i. with MPEC strains *E. coli* P4 and 1303, and non-MPEC strains *E. coli* K12 strains DH5alpha and MG1655 compared to PBS controls. **(C)** Association of IL-8 levels and bacterial loads in mammary tissue at 24 h p.i. with MPEC strains *E. coli* P4 and 1303, and non-MPEC strains *E. coli* K12 strains DH5alpha and MG1655 (*n* ≥ 7; *r* = 0.63; *P* < 0.01; 95% C.I. 0.354–0.808). **(D)** CHI3L1 levels in protein lysates extracted from mammary tissue at 24 h p.i. with MPEC strains *E. coli* P4 and 1303, and non-MPEC strains *E. coli* K12 strains DH5alpha and MG1655 compared to PBS controls (*n* ≥ 8). Data are presented as means ± SEM, indicating significant differences with *(*P* < 0.05); **(*P* < 0.01) or ***(*P* < 0.001). ns, not significant.

The observations in coliform-infected dairy cows were mimicked by experimentally infecting the mammary gland of C57BL/6 mice (inoculum dose = 10^3^ CFU) with two *E. coli* strains P4 and 1303, isolated from acute cases of bovine mastitis, both belonging to phylogroup A (37, 38). Two non-MPEC *E. coli* strains (i.e., K-12 strains DH5alpha and MG1655) that also belong to phylogroup A, were inoculated at the same dose and alongside the MPEC *E. coli* strains P4 and 1303. At 24 h p.i., high bacterial loads were observed in the mammary glands with significantly different bacterial counts between *E. coli* strain P4 and 1303 infected glands (9.64 ± 0.26 and 7.84 ± 1.70 log_10_ CFU/g mammary gland, respectively) (Figure 2A). Both these non-MPEC strains yielded significantly lower bacterial loads compared to the two MPEC strains. Mammary gland infection with both MPEC strains was characterized by a high influx of immune cells in the alveolar lumen compared to PBS (sham) inoculated glands, whereas both non-MPEC strains also induced an immune cell recruitment albeit limited compared to the two MPEC strains (Figure 2B). Comparison of bacterial loads and IL-8 levels showed that *E. coli* strains with high bacterial loads correlated with mammary IL-8 levels (Pearson’s correlation *r* = 0.63, *P* < 0.01, 95% C.I. 0.354–0.808) (Figure 2C). This strongly positive correlation can be interpreted as a tendency for high CFU values that go with high IL-8 levels (and *vice versa*). In marked contrast to the latter observation, similar mammary CHI3L1 levels were observed after infection with either MPEC or non-MPEC strains compared to PBS inoculated (sham) mammary glands (Figure 2D).

### CHI3L1 Is Required for Neutrophil Influx in the Alveolar Lumen and Influences a Selected Panel of Mammary Cytokines in the Mouse *E. coli* Model for Mastitis

**Figure 3:**
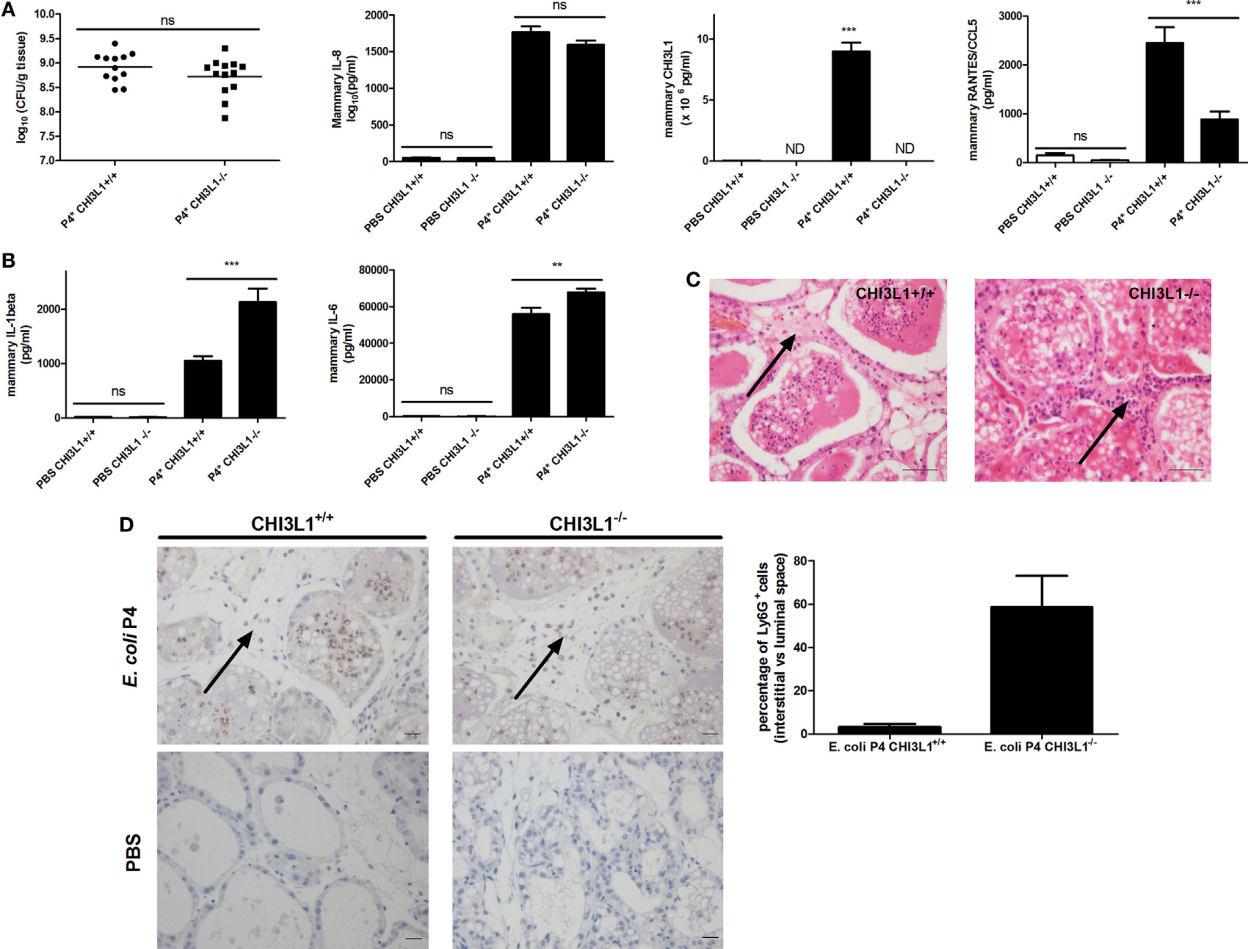
Downstream effects of mammary chitinase 3-like 1 (CHI3L1) during *Escherichia coli* P4 infections in the mouse mastitis model. **(A)** Bacterial loads (*n* ≥ 12), and interleukin (IL)-8 (*n* ≥ 3), CHI3L1 (*n* ≥ 5), and RANTES/CCL5 levels (*n* ≥ 3) in the mammary glands of CHI3L1^+/+^ and CHI3L1^−/−^ mice 24 h p.i. with *E. coli* P4. **(B)** IL-1beta (*n* ≥ 3) and IL-6 levels (*n* ≥ 3) in the mammary gland of CHI3L1^+/+^ and CHI3L1^−/−^ mice 24 h p.i. with *E. coli* P4. **(C)** H&E histology of mammary tissue 24 h p.i. with *E. coli* P4 in CHI3L1^+/+^ and CHI3L1^−/−^ mice. Black arrow illustrates the presumed neutrophilic innate immune cells (based on their characteristic polymorphonuclear morphology) in the interstitial space. **(D)** Immunohistochemical stainings for Ly6G on paraffin slides of mammary glands at 24 h p.i. with *E. coli* P4 or phosphate buffered saline (PBS) in CHI3L1^+/+^ and CHI3L1^−/−^ mice. Black arrows indicate Ly6G-positive neutrophils in the interstitial space. Scale bars = 20 µm. The bar graphs display the quantified percentage of Ly6G-positive cells in the interstitial space versus the luminal space for stained sections of *E. coli* P4-infected mammary glands from CHI3L1^+/+^ and CHI3L1^−/−^ mice (*n* ≥ 2 glands). Data are presented as means ± SEM, indicating significant differences with **(*P* < 0.01) or ***(*P* < 0.001). ns, not significant.

Current literature on CHI3L1 in the mammary gland focuses on its role during breast cancer development. In order to decipher its immunological role during mammary infection, CHI3L1 knock-out (CHI3L1^−/−^) and wild-type (CHI3L1^+/+^) animals were at first intramammarily inoculated with either PBS or *E. coli* P4 (Figure 3A). Bacterial loads, IL-8, CHI3L1, and RANTES/CCL5 levels were determined in CHI3L1^−/−^ versus CHI3L1^+/+^ mice. At 24 h p.i., bacterial loads and IL-8 levels significantly increased in the mammary gland independent of CHI3L1 in clear contrast to mammary RANTES/CCL5 levels that were significantly reduced in CHI3L1^−/−^ compared to CHI3L1^+/+^ mice (Figure 3A). Moreover, CHI3L1 also influenced the levels of some other pro-inflammatory cytokines. In contrast to RANTES/CCL5, IL-1beta and IL-6 protein levels in mammary tissue homogenates were significantly increased at 24 h p.i. in CHI3L1^−/−^ mice compared to CHI3L1^+/+^ mice (Figure 3B). Histology showed striking differences between CHI3L1^−/−^ and CHI3L1^+/+^ mammary glands at 24 h p.i. (Figure 3C). Immunostaining against Ly6G, a marker predominantly present on neutrophils, showed that neutrophils infiltrated the lumen of the mammary alveoli of the infected CHI3L1^−/−^ mice (Figure 3D). However, the majority of these innate immune cells were still present in the interstitial space between the alveoli. This key observation was in marked contrast to infected CHI3L1^+/+^ glands where only a limited number of immune cells could be observed in the interstitial space and the majority was present in the alveolar lumen (Figure 3D).

### Pan-Caspase Inhibition and Chitin Treatment Decrease Mammary CHI3L1 During *E. coli* P4 Infection in a Mouse Mastitis Model

**Figure 4:**
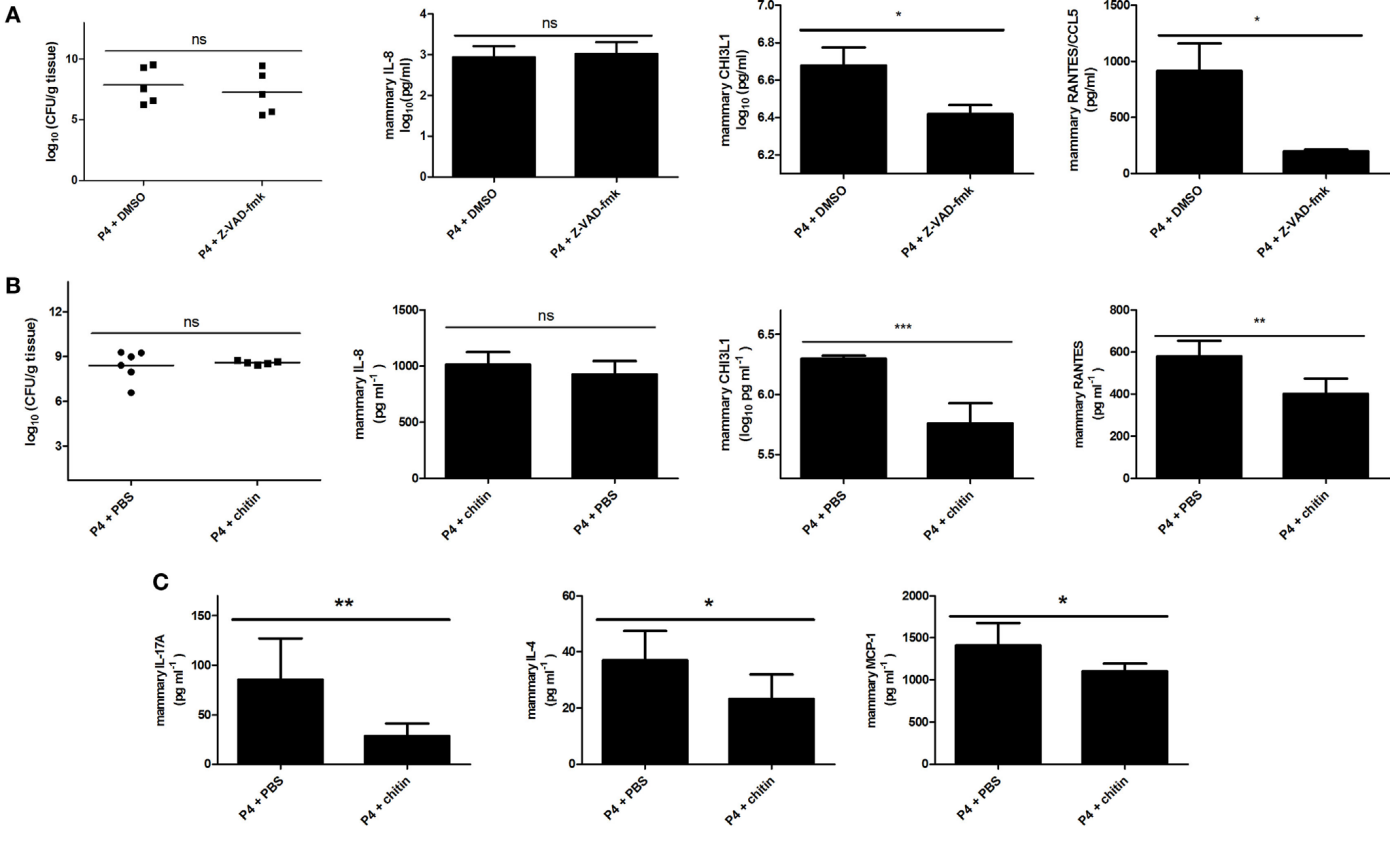
Upstream regulators of mammary chitinase 3-like 1 (CHI3L1) during *Escherichia coli* P4 infections in the mouse mastitis model. **(A)** Bacterial loads (*n* = 5), and interleukin (IL)-8 (*n* = 5), CHI3L1 (*n* ≥ 4), and RANTES/CCL5 levels (*n* = 5) in the *E. coli* P4-infected mammary glands of mice treated with Z-VAD-fmk or DMSO (sham) at 24 h p.i. **(B)** Bacterial loads (*n* ≥ 12), and IL-8 (*n* ≥ 8), CHI3L1 (*n* = 6), and RANTES/CCL5 levels (*n* = 6) in the mammary gland of mice treated with chitin or phosphate buffered saline (PBS) controls at 24 h p.i. with *E. coli* P4. **(C)** IL-17A (*n* = 6), IL-4 (*n* = 6), and MCP-1 levels (*n* = 6) in the *E. coli* P4-infected mammary gland of mice treated with chitin or PBS controls at 24 h p.i. Data are presented as means ± SEM, indicating significant differences with *(*P* < 0.05); **(*P* < 0.01); or ***(*P* < 0.001). ns, not significant.

**Figure 5:**
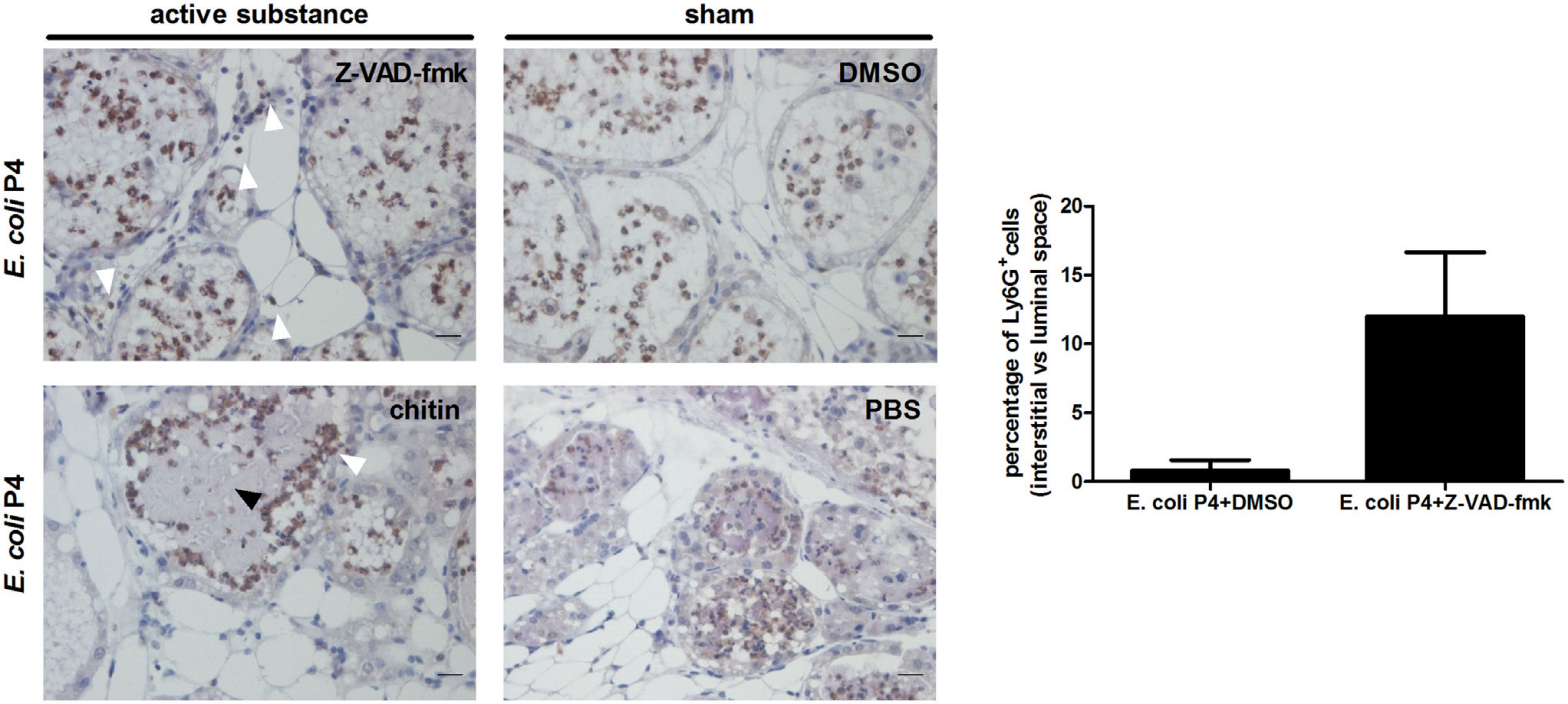
Ly6G immunohistochemistry in *Escherichia coli* P4-infected mammary glands posttreatment with active substances or sham controls. Immunohistochemical stainings for Ly6G on paraffin slides of *E. coli* P4-infected mammary glands treated with Z-VAD-fmk, DMSO, chitin, or phosphate buffered saline (PBS). White arrows indicate Ly6G-positive neutrophils. Black arrows indicate bacterial clusters. Scale bars = 20 μm. The bar graph displays the quantified percentage of Ly6G-positive cells in the interstitial space versus the luminal space for stained sections of *E. coli* P4-infected mammary glands treated with DMSO or Z-VAD-fmk (*n* = 2 glands). Data are presented as means ± SEM.

Our data are in accordance to previous observations stating that IL-1beta signaling influences neutrophil trapping in the interstitial space of the infected mammary gland (39). To assess the effect of IL-1beta activators, such as caspase-1 and caspase-11, on CHI3L1 in the mammary gland, mice were intramammarily inoculated with *E. coli* P4 and then treated with a single bolus of either the pan-caspase inhibitor (Z-VAD-fmk) or DMSO (sham) administered *via* the same route at 4 h p.i. As indirect parameter supporting the (partly) inhibitory effect of local Z-VAD-fmk treatment on caspase function, a TUNEL assay was performed on mammary gland sections of wild-type mice intramammarily infected with *E. coli* P4 (Figure S4 in Supplementary Material). Histology of these *E. coli*-infected mammary glands treated with Z-VAD-fmk contained on average 47% less TUNEL-positive cells compared to the sham-treated mammary glands condition. Mammary CHI3L1 levels significantly decreased upon pan-caspase inhibition compared to DMSO (sham) treatment, independently of bacterial loads and IL-8 levels at 24 h p.i. (Figure 4A). Concomitantly, RANTES/CCL5 levels were also significantly decreased at 24 h p.i. (Figure 4A). However, the decrease in CHI3L1 after treatment with Z-VAD-fmk was not strong enough to induce an effective neutrophil trapping. Indeed, the presence of Ly6G-positive cells in the interstitial space of mammary glands infected with *E. coli* was less evident following the Z-VAD-fmk treatment (white arrows in Figure 5) compared to clear observations in the CHI3L1^−/−^ mice. Nonetheless, in our DMSO control (sham) no interstitial immune cells could be visualized (Figure 5).

Intraperitoneal administration of chitin particles to mammary tumor-bearing mice has been demonstrated to significantly reduce local CHI3L1 levels (10). To test the effect of local chitin particle treatment on CHI3L1 induction during experimental mastitis, mammary glands were inoculated with *E. coli* P4 (inoculum dose = 10^3^ CFU), followed by intramammary application of either chitin particles or PBS (sham) 4 h p.i. Mammary CHI3L1 and RANTES/CCL5 levels significantly decreased upon chitin treatment compared to PBS (sham) treatment, independently of bacterial loads and IL-8 levels at 24 h p.i. (Figure 4B). Mammary levels of selected pro-inflammatory cytokines IL-17A, IL-4, and MCP-1/CCL2 also significantly decreased upon chitin treatment compared to PBS (sham) treatment at 24 h p.i. (Figure 4C). Although chitin treatment had a larger impact on the cytokine profiles in comparison to caspase inhibition, the Ly6G-positive cells were still able to cross the epithelial barrier (Figure 5). However, these migrated neutrophils were rather observed on the extremities of the alveolar lumen (white arrows in Figure 5) and more specifically around bacterial clusters (black arrows in Figure 5) but were absent in our PBS (sham) controls (Figure 5).

In addition to its inhibitory effect on the main chitinase-like family member (CHI3L1), chitin treatment also decreased the mammary chitinolytic activity at 24 h p.i. (Figures S2B,C in Supplementary Material). The *E. coli* P4-infected glands showed a significant increase in chitinolytic activity as measured both with *N,N*′-diacetyl-β-d-chitobiose and *N,N′,N′′-*triacetyl-β-d-chitotriose substrates compared to the PBS (sham) inoculated mammary glands (Figure S2B in Supplementary Material). However, following chitin particle treatment, the fluorescent signal produced by cleavage of both the 4-MU *N,N*′-diacetyl-β-d-chitobiose and 4-MU *N,N′,N′′-*triacetyl-β-d-chitotriose significantly decreased at 24 h p.i. (Figure S2C in Supplementary Material).

Finally, it was also verified whether the observed inhibitory effect of chitin treatment on local CHI3L1 induction was mouse strain-dependent. Mammary glands of CD-1 outbred mice were infected with *E. coli* P4 and subsequently treated with either chitin particles or PBS (sham) at 4 h p.i. Again, a significant decrease in mammary CHI3L1 levels was observed as a consequence of the chitin particle treatment compared to a PBS (sham) treatment (Figure S3B in Supplementary Material). Again, this decrease was dependent on the dose of chitin used but not related to the bacterial loads (Figure S3A in Supplementary Material). Additionally, chitin treatment of *E. coli* P4 infected animals again decreased the exo- and endochitinase activities (Figure S3C in Supplementary Material).

## Discussion

Induction of inflammation is a crucial initializing step in bovine coliform mastitis (40, 41). Nonetheless, the contribution of both host and bacterial factors to initiation, progression and resolution of inflammation remains poorly characterized. Among proteins receiving recent attention are inflammatory mediators, such as CHI3L1, and its regulatory role in the host innate immune response are increasingly studied.

A wide variety of host innate immune cells, including neutrophils, monocytes/macrophages, and some epithelial cell lines can either express CHI3L1 or are affected by this regulatory protein through its binding to IL-13 Receptor α2 (18, 21, 42–47). As coliform mastitis involves the interplay of resident and infiltrating innate immune cells, our observations of significantly increased CHI3L1 protein levels and CHI3L1 transcription in both udders natural and experimental infected with *E. coli* compared to healthy quarters are in line with these data. Nevertheless, CHI3L1 has been only scarcely reported in some proteomic studies on bovine mastitis samples obtained from either natural or experimentally coliform-infected cows (27–29). As these studies are typically descriptive, the function of chitinase(-like) proteins in the context of bovine mastitis remains largely unclear.

Strains belonging to *E. coli* phylogroup A are most frequently isolated from coliform mastitis cases (48). Therefore, we used here the mouse mastitis model to mimic the marked induction of mammary CHI3L1 in cows by performing experimental intraductal infections with two bovine phylogroup A mastitis isolates, *E. coli* P4 and 1303 (49, 50). Interestingly, CHI3L1 was equally induced at 24 h p.i. by both these MPEC strains, although infection with *E. coli* P4 yielded significantly higher bacterial loads, IL-8 levels, and subsequent neutrophil recruitment compared to *E. coli* 1303 infection. Two non-MPEC strains of phylogroup A (K-12 strains DH5alpha and MG1655) yielded significantly lower bacterial loads and concomitant IL-8 levels at 24 h p.i. compared to the two MPEC strains. Again, infection status had a marked correlation with IL-8, but not CHI3L1 levels were seen between the four *E. coli* infection groups.

To subsequently further assess potential molecules co-regulated by/with CHI3L1, we next used CHI3L1-deficient mice in the context of mammary gland infection to corroborate our observations. Similar to our findings using whey and mammary tissue, we demonstrated a remarkable trapping of neutrophils in the interstitium of *E. coli* P4-infected CHI3L1^−/−^ mice, which was associated with an aberrant induction of the pro-inflammatory mediator RANTES/CCL5. Nevertheless, although the latter cytokine is known as a leukocyte recruiter, its precise function in the mammary gland is currently unknown (51). Interestingly, we observed further that the lack of the CHI3L1-RANTES/CCL5 axis was associated with a significant increase of the two key pro-inflammatory cytokines IL-1beta and IL-6. These data are in agreement with the observation that the lack of IL-1 receptor signaling is related to an inefficient neutrophil recruitment into the alveolar lumen during endotoxin-induced mastitis (39, 52). Furthermore, a lack of CHI3L1 has been reported to be responsible for an increase of IL-1beta during lung infection in mouse models (15, 16). CHI3L1 has been shown to contribute to the facilitation of bacterial invasion into the intestinal mucosa as well as to the development of acute colitis with *E. coli* strains distinct from mastitis strains (53). Thus, when carefully extrapolating such a direct effect of CHI3L1 to the context of *E. coli* mammary gland infections, targeting the CHI3L1 signaling pathway may open new avenues for future research regarding the modulation of the host immune response to coliform mastitis. Our findings on the immunomodulatory function of mammary CHI3L1 complement the previously described function of a related mammalian CLP, stabilin-1 interacting CLP, that binds endotoxin and neutralizes the effect of this major *E. coli* virulence factor on the hosts’ tissue (54).

To gain insight in the signaling of CHI3L1 in the mammary gland, we subsequently explored the regulatory aspects of this protein. At 1 day after experimental MPEC mammary infection, local IL-1beta levels significantly increased in CHI3L1^−/−^ compared to CHI3L1^+/+^ mice. As the classical maturation of pro-IL1beta to IL-1beta occurs *via* caspase-1, this key finding suggests a possible link between CHI3L1 and caspase activation in the *E. coli*-infected mammary gland. Supporting this hypothesis, we previously reported a non-classical, caspase-independent cleavage of pro-IL1beta to IL-1beta in the mammary gland of *E. coli* P4 infected CHI3L1^+/+^ mice (55). In line with our current observation in CHI3L1-deficient mice, a *Streptococcus pneumoniae* infection also induced higher levels of IL-1beta in BAL fluid of CHI3L1^−/−^ compared to CHI3L1^+/+^ mice (15). The latter authors linked their finding to the CHI3L1-dependent regulation of a caspase-1 and Nlpr3 inflammasome, or *vice versa* (15). Therefore, a pan-caspase inhibitor was subsequently used to identify at first the probable location of caspases in the CHI3L1 signaling during coliform induced mammary inflammation in mice. This information would inform us whether CHI3L1 is responsible for the neutrophil influx into the alveolar lumen independently of IL-1beta signaling previously reported (39). The observation that the number of cells containing double-stranded DNA breaks was reduced due to local Z-VAD-fmk treatment, is indicative for the fact that *in vivo* apoptosis was affected by this pan-caspase inhibitor (56). As this reduction is an indirect read-out for a decreased activity of apoptotic caspases, it can be further assumed that also caspase-1 and -11, both mediators of pro-IL1beta maturation, might—at least partly—have been inhibited by Z-VAD-fmk. Furthermore, Ly6G immunohistology data showed less prominent neutrophil trapping compared to our observations with CHI3L1^−/−^ mice. Together, these findings imply that both IL-1beta and CHI3L1 are important for the neutrophils to cross the epithelial barrier during mammary inflammation.

Alternatively, CHI3L1 levels can also be modulated through interaction with chitin. The detailed inhibitory mechanism of CHI3L1 through chitin remains to be unraveled, it is known that chitin selectively and strongly binds to CLPs through a carbohydrate-binding motif (CBM) in its C-terminus (57). The loss of this CBM motif or a mutation herein either eliminates or decreases CHI3L1-induced cell signaling in colonic epithelial cells (57, 58). This mechanism was also suggested through the *in vivo* blockage of the CBM with a specific anti-CHI3L1, which facilitated dextran sulfate sodium-induced colitis (53). In that belief, we instilled chitin particles directly into the target tissue to block the CBM of local CHI3L1 in the mastitic mouse mammary gland. This immunomodulation concept was adapted from a recent breast cancer report that also could lower local CHI3L1 levels, albeit through the intraperitoneal administration of chitin particles in mammary tumor-bearing mice (10). Our experiments now extend these data indicating that, similar as seen with the pan-caspase inhibition, local chitin particle treatment as expected significantly reduces the mammary levels of CHI3L1 but more importantly also causes less activation of local RANTES/CCL5. However, these changes did not affect bacterial loads nor local activation of IL-8. In comparison to the observations in CHI3L1^−/−^ mice, the influx of neutrophils into the luminal space was less affected compared to caspase inhibitor or chitin treatment. The former treatment is in line with our previous observations that caspase activity is only partly responsible for proIL-1beta maturation in the inflamed mammary gland (55). Moreover, the trapping of immune cells observed in inflamed mammary glands of IL-1beta^−/−^ animals is also less evident (39). Interestingly, although interstitial neutrophils could still cross the epithelial barrier after pretreatment with chitin, their influx was disturbed and led to bacterial clusters embedded in these neutrophils. This observation may be indicative for the fact that chitin has another mechanism than mere inhibition of CHI3L1.

In the current study, local chitin particle treatment was observed to lower also the mammary chitinolytic activity in our mouse model for bovine coliform mastitis. These additional chitin-binding proteins were not considered in the mammary tumor paper (10). We demonstrated at first that both in cows and in mice the chitinase activity is induced by coliform mastitis. In the cow genome, the G18 family consists of three chitinases, i.e., CTBS, AMCase, and CHIT1 (4). CTBS is an exochitinolytic member cleaving the disaccharide *N,N′*-diacetyl-β-d-chitobiose and the trisaccharide *N,N′,N′*-triacetyl-β-d-chitotriose of GlcNAc polymers (59). The *in vivo* function of CTBS activity in the mammary gland is unclear. It has been suggested to have a general immunological function through cleavage of target glycosylated proteins in lysosomes, but it also modified glycoproteins in breast milk and in a bovine kidney cell line (60–62). The two other chitinases, CHIT1 and AMCase, have endochitinolytic activity and are, therefore, able to digest natural chitin as well as artificial chitobiose and chitotriose substrates (63). The endochitinase activity was predominantly studied in airway pathologies because the major chitinolytic activity is observed in the lung. Intranasal delivery of chitin induced an accumulation of immune cells which was mainly dependent on AMCase (64), while an increased asthma risk is suggested to be primarily the consequence of CHIT1 polymorphisms (11).

In analogy with CHI3L1, chitinases also contain a CBM. Currently, the consequences of inhibiting CBM-induced signaling in chitinases are still poorly understood. Alternative splicing observed in human macrophage CHIT1 resulted in a truncated isoform lacking the CBM which was unable to bind chitin, but was still able to hydrolyze chitotriose (65). In line with these data, we speculate that CBM signaling—both in the non-lytic chitin-binding proteins like CHI3L1 and in the chitinases—is blocked by the chitin particle treatment rather than a direct inhibition of chitinolytic activity. Modulation upon chitin treatment of the cytokine profile—additional to the one associated with the CHI3L1-RANTES/CCL5 axis—would then be a result of the reduced chitinase signaling. Moreover, chitin and AMCase have been shown to influence macrophage activation and the accumulation of IL-4 expressing innate immune cells in allergy models (64). Chitinases enhanced MCP-1 and increased the migratory capacity of macrophages in a blood–brain barrier *in vitro* model. Finally, it should be remarked that RANTES/CCL5 is also known to be induced by chitinases (66, 67), while CHI3L1 can induce macrophage recruitment by MCP-1 chemotaxis in colorectal cancer and has a role in IL-17A-dependent mechanisms (68, 69).

In conclusion, immunomodulatory treatments targeting to alter the levels and/or function of local chitinase(-like) proteins may offer a novel strategy to reduce the use of antibiotics in the dairy industry especially in the field of *E. coli* mammary gland infections, where antibiotic treatment remains the major intervention to date (70, 71). Our study partly unraveled some innovative aspects of this immunomodulation axis of CHI3L1 during coliform mammary infection. As CHI3L1 has an important Th2 modulating immunity function through IL-13 Rα2 (33, 47), it is likely that this protein is also of key importance in later time points post-infection with either *E. coli* or even with chronic mastitis pathogens. Interestingly, IL-13—a cytokine that co-localizes with CHI3L1, the IL-13 Rα2, and transmembrane protein TMEM219 to participate in a multimeric complex (47, 72)—was not detected in our short experimental timeframe (data not shown), while staphylococcal enterotoxins responsible for chronic infections have been reported (53). Future research exploring in-depth family members with chitinase enzymatic activity and other CLPs will further elucidate the mechanisms of inflammation in the infected mammary gland.

## Ethics Statement

This study was carried out in accordance with the FELASA guidelines and recommendations. The protocol was approved by the Committee on the Ethics of Animal Experiments of the Ghent University.

## Author Contributions

KB, JS, KD, and EM were important for conception and design of the study, analysis and interpretation of the data, as well as drafting of the manuscript. KB, JS, KD, WP, and PG contributed to the acquisition of the data. DS, PG, and EM revised the manuscript critically. KB, JS, KD, WP, DS, PG, CGL, JAE, and EM gave their approval of the final manuscript to be published.

## Conflict of Interest Statement

The authors declare that the research was conducted in the absence of any commercial or financial relationships that could be construed as a potential conflict of interest.
