# Supplementary figures and images for: Immunomodulation of Host Chitinase 3-Like 1 During a Mammary Pathogenic Escherichia coli Infection
Source: Front Immunol. 2018 May 28;9:1143. doi: 10.3389/fimmu.2018.01143 (PMC5985307; doi:10.3389/fimmu.2018.01143)

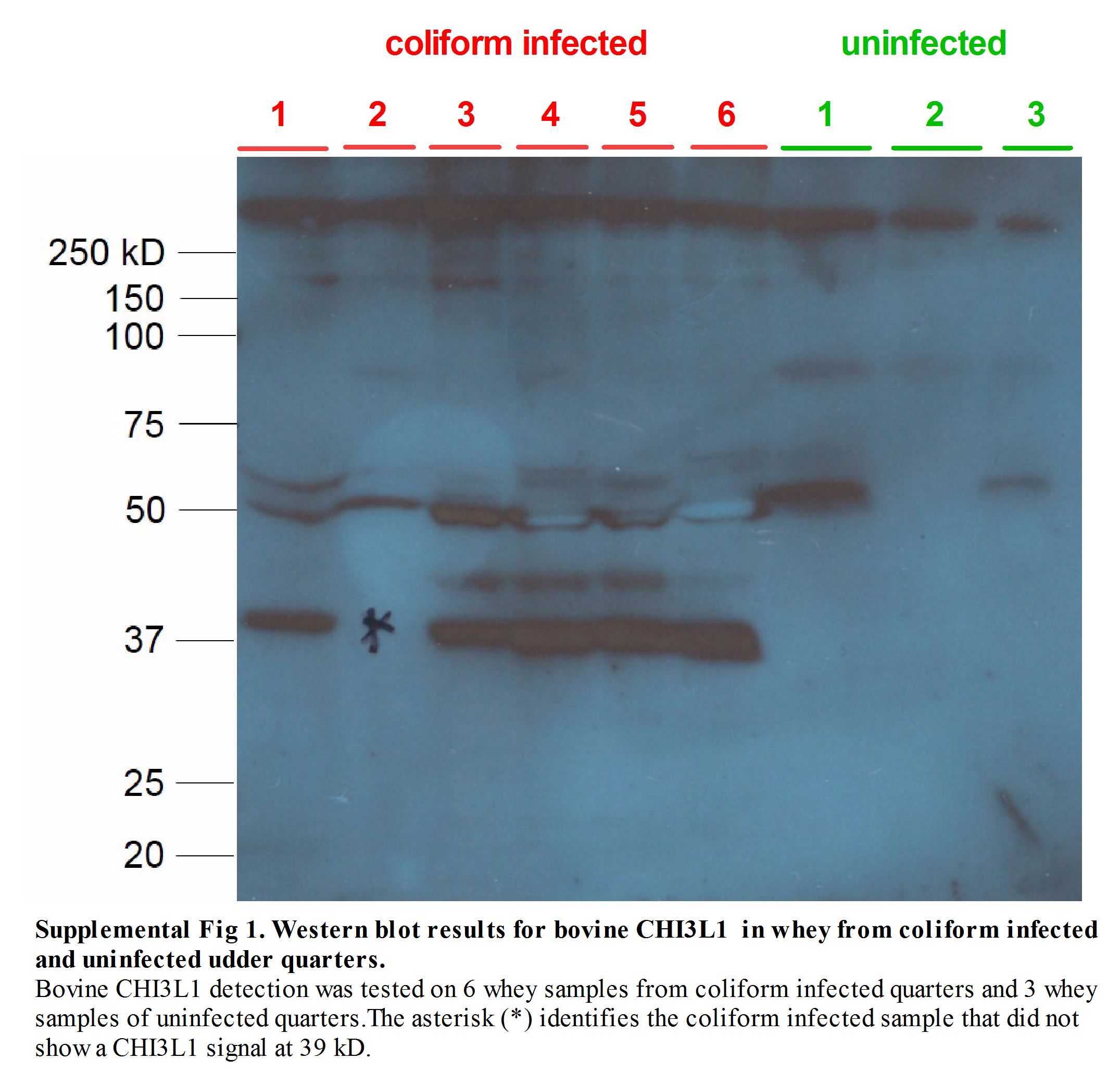

Supplement: Supplementary file 1 [file image_1.tif]

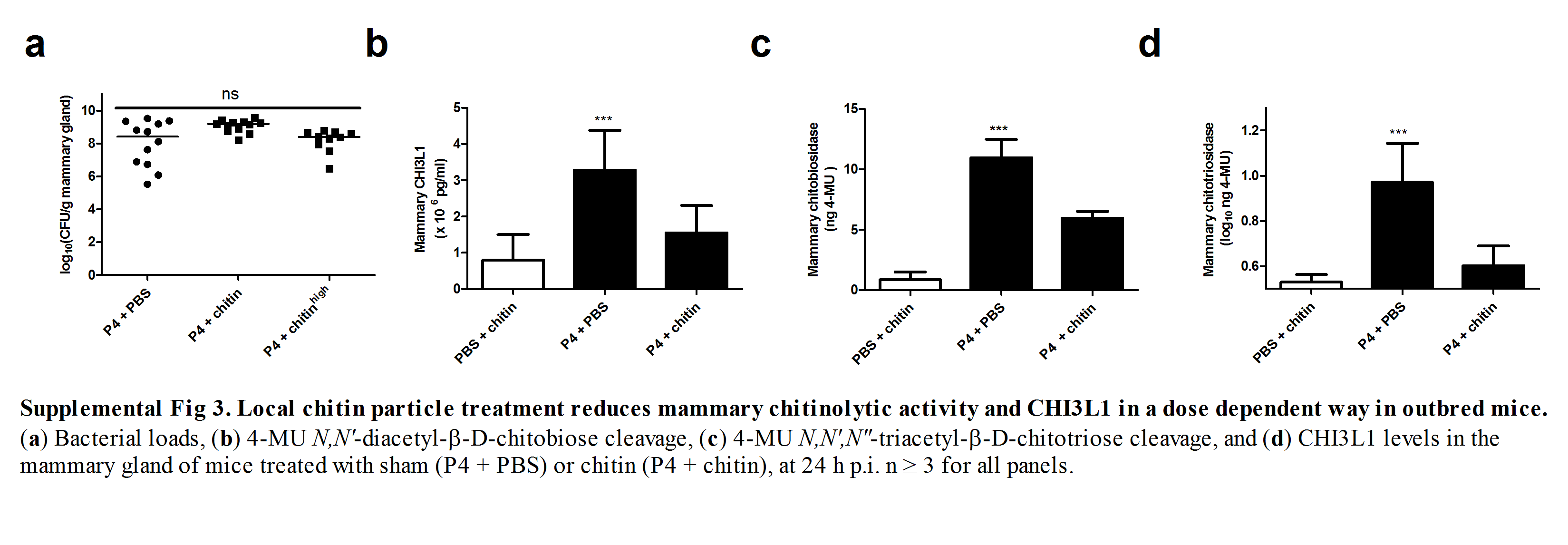

Supplement: Supplementary file 3 [file image_3.tif]

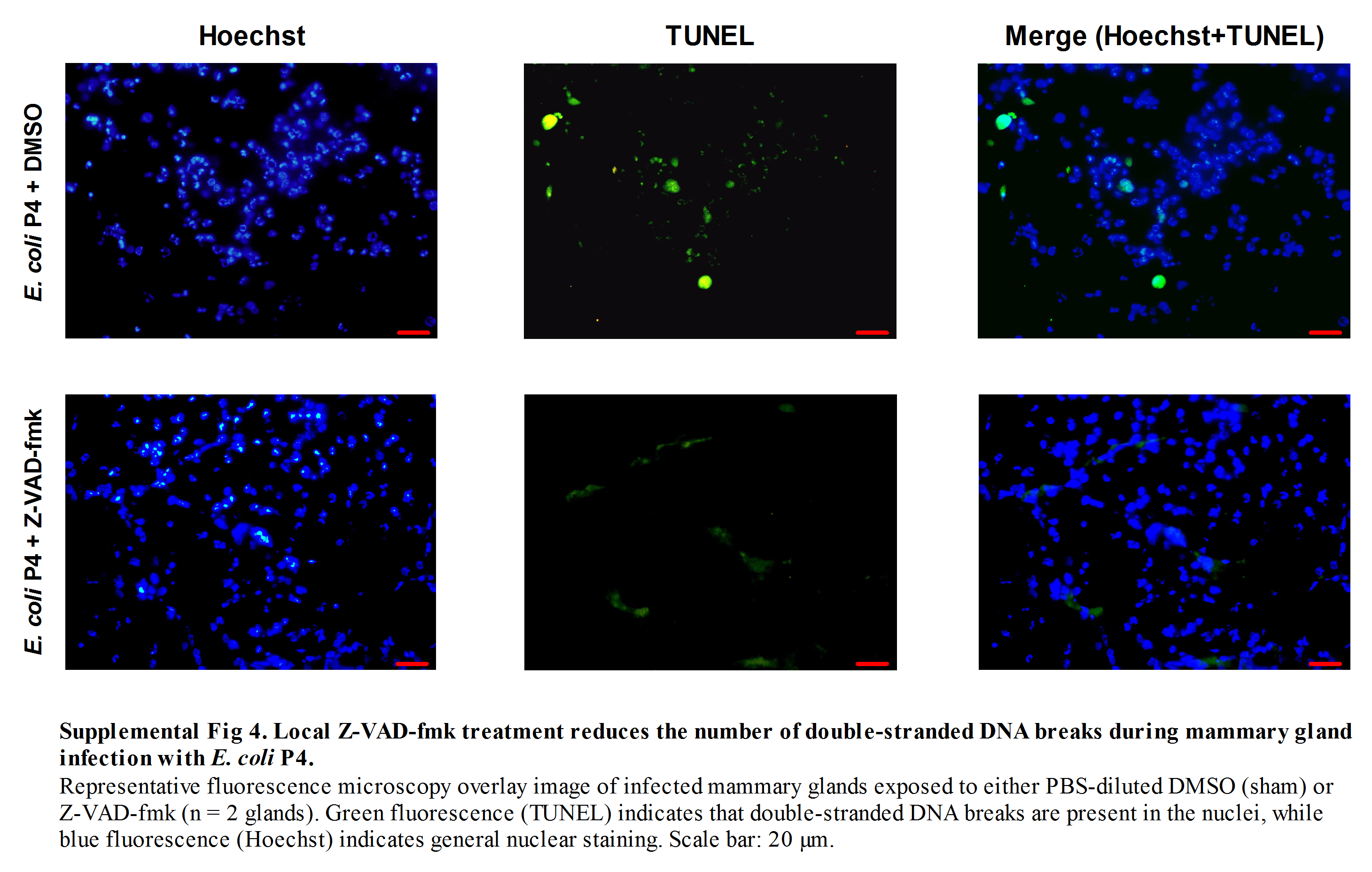

Supplement: Supplementary file 4 [file image_4.tif]
